# Impact of metal ions on PCR inhibition and RT-PCR efficiency

**DOI:** 10.1007/s00414-020-02363-4

**Published:** 2020-07-03

**Authors:** Agnieszka Kuffel, Alexander Gray, Niamh Nic Daeid

**Affiliations:** Leverhulme Research Centre for Forensic Science, University of Dundee, Dundee, Scotland

**Keywords:** PCR inhibition, Metal ions, DNA polymerase, EGTA

## Abstract

Inhibition of PCR by metal ions can pose a serious challenge in the process of forensic DNA analysis. Samples contaminated with various types of metal ions encountered at crime scenes include swabs from metal surfaces such as bullets, cartridge casings, weapons (including guns and knives), metal wires and surfaces as well as bone samples which contain calcium. The mechanism behind the impact of metal ions on DNA recovery, extraction and subsequent amplification is not fully understood. In this study, we assessed the inhibitory effects of commonly encountered metals on DNA amplification. Of the nine tested metals, zinc, tin, iron(II) and copper were shown to have the strongest inhibitory properties having IC50 values significantly below 1 mM. In the second part of the study, three commercially available DNA polymerases were tested for their susceptibility to metal inhibition. We found that KOD polymerase was the most resistant to metal inhibition when compared with Q5 and Taq polymerase. We also demonstrate how the calcium chelator ethylene glycol-bis(2-aminoethylether)-N,N,N′,N′-tetraacetic acid (EGTA) can be used as an easy and non-destructive method of reversing calcium-induced inhibition of PCR reactions.

**Electronic supplementary material:**

The online version of this article (10.1007/s00414-020-02363-4) contains supplementary material, which is available to authorized users.

## Introduction

The effect of metal ions on DNA and PCR amplification is not fully understood, despite there being a significant number of studies that focus on the inhibitory properties of metals. The published research shows that metal ions can interfere with DNA analysis at various points; from extraction to PCR amplification [1–6] and as a result hinder subsequent DNA profiling. The impact of the interference is heavily dependent on the concentration and the metal involved. It is assumed that metal ions have an affinity for DNA mediated by the negative charge on the phosphate back bone of the DNA interacting with the positively charged metal ions. Some metals such as K^+^, Na^+^ and Mg^2+^ are essential for DNA stability [7]. Many interactions between metal ions and DNA are essential for the correct functions of DNA related enzymes. However despite their crucial role, under certain conditions, these interactions can still lead to adverse effects on DNA outside as well as inside the human body [8].

The presence of metal ions in a biological sample can originate from various sources for example, calcium from bone and iron from blood. Contamination of DNA samples by metal ions may also originate from the environment when the sample is collected from metal surfaces such as wires, cartridge cases or metal gun parts [9–12]. Analysis of DNA samples extracted from bones can be particularly challenging due to the inhibitory effect of calcium on PCR amplification [13]. Calcium has been found to be a Taq polymerase inhibitor, competitively binding to the polymerase in place of magnesium during PCR and as a result reducing the efficiency of amplification [2, 3]. Ammunition is already quite a challenging surface for DNA recovery as on top of metal inhibition, firing bullets has been found to decrease the amount of DNA recovered from their surface by about 30% [14]. Tin is ubiquitous in everyday items such as food packaging and beverage containers [15] that can be a source of touch DNA. Metal wires and cables [16–18] are often the subject of theft from buildings and equipment. Improvised explosive devices (IEDs) contain copper wires which may also be a source of touch DNA [19–21]. Recent work regarding DNA recovery from explosive devices has shown that plasticisers may have a strong inhibitory effect on DNA amplification and may be as problematic as metal inhibition [22].

In the case of archaeological bone samples that already contain calcium, additional metal ions can also be transferred from artefacts surrounding bone fragments. Those objects can include various tools, pieces of jewellery or clothing usually made out of copper, bronze or brass [4]. However at the same time, these metal ions may protect samples from microbial growth and enhance specimen preservation [23]. Metals may also leach directly from the surrounding soil [4]. Furthermore, the humic substances found in soil have the ability to bind metal ions [24] and form soluble, high molecular weight complexes with DNA which can lead to impaired access to the DNA template and as a result, reduce the efficiency of PCR amplification. In addition, some metals are known to produce extensive crosslinks between DNA and proteins. Examination of in vivo crosslinking of proteins and DNA caused by heavy metals, Wedrychowski et al. determined that mercury, copper, lead and aluminium had the ability to crosslink some nuclear proteins to DNA [25]. Even though the study was conducted in living cells, crosslinking may also occur in biological samples and explain some of the mechanisms behind metal ion-DNA interactions and their impact on forensic DNA analysis. Through formation of direct crosslinks with DNA, metal ions can easily inhibit PCR by blocking access to the DNA template. Examples of interactions between metals and DNA include aluminium forming at least three crosslinks with DNA [26] and copper showing high affinity binding to DNA bases [27]; nickel binding to DNA has been found to be sequence and pH specific [26] with lead showing some sequence-specific, tight DNA binding [28].

The majority of PCR inhibitors impact PCR results in one of three ways: (i) by interfering with cell lysis required for DNA extraction; (ii) by degrading or capturing the nucleic acid; and (iii) by inhibiting activity of DNA polymerases [6]. However, poor DNA analysis results from metal objects may be caused by more than just PCR inhibition during amplification [29]. In some cases, DNA loss may occur at much earlier stages, before the recovery process even begins, for example, DNA left on surfaces containing copper, such as cartridge casings, is thought to be susceptible to damage resulting from contact with the metal surface itself [29].

When collecting DNA samples from various metal surfaces, contamination of the sample with inhibitory metal ions is often unavoidable. Although they may be removed during the process of DNA extraction, co-purification of PCR inhibitors during DNA extraction is a very common occurrence [30–32] and can have a negative effect on DNA analysis [33].

The aim of this study was to systematically observe how amplification of DNA can be affected by presence of metal ions. In order to focus on the amplification step only, we decided to use naked DNA of known concentration thereby omitting the extraction steps. As a consequence, we are aware of the limitations that this study presents when applying the results to real-life samples. However, the data presented does provide relevant comparative information on which metals are likely to prove more or less problematic as inhibitors of PCR reactions.

In this study, we have tested the concentration dependence of metal ion inhibition of PCR as well as the susceptibility of different DNA polymerases to metal inhibition. We also demonstrate a simple way of removing calcium ion inhibition.

## Materials and methods

Metal stock solutions (40 mM) of following metals were prepared in water: copper(II) sulfate (copper(II) sulfate, puriss. p.a., anhydrous ≥ 99%, Sigma-Aldrich), iron(II) sulfate (Ferrous sulfate, dried, VWR), aluminium sulfate (aluminium sulfate hexadecahydrate, Extra Pure, SLR, Fisher Chemical), nickel(II) sulfate (nickel(II) sulfate hexahydrate, 99%, for analysis, ACROS Organics™, Thermo Fisher Scientific), iron(III) chloride (iron(III) chloride hexahydrate, 99+%, for analysis, ACROS Organics™, Thermo Fisher Scientific), lead(II) nitrate (lead(II) nitrate, 99+%, Technical, Fisher Chemical), tin(II) chloride (tin(II) chloride, 98%, anhydrous, ACROS Organics™, Thermo Fisher Scientific), zinc chloride (zinc chloride reagent grade, ≥ 98%, Sigma-Aldrich) and calcium chloride (calcium chloride, 96%, extra pure, powder, anhydrous, ACROS Organics™, Thermo Fisher Scientific).

### Agarose gel electrophoresis

DNA analysis by agarose gel electrophoresis was carried out on 1% w/v Agarose (Thermo Fisher Scientific molecular biology grade) gels containing TAE buffer (40 mM Tris Base, 20 mM Acetic acid, 1 mM EDTA pH 8.3) and 1× Sybr Safe Gel Stain (Thermo Fisher Scientific) added from the manufacturers 10,000× stock as supplied. Gels were run submerged in TAE buffer at 120-V constant voltage for 35–40 min. DNA bands were photographed under blue light using a Fujifilm LAS3000 camera system. The detection limit for this system is 250 pg of DNA per band.

### qPCR

Each test sample contained TaqMan™ Control Genomic DNA (Thermo Fisher Scientific) at the indicated concentrations as listed in the results. All the reactions were run with Applied Biosystems™ PowerUp™ SYBR™ Green Master Mix (Thermo Fisher Scientific) in 10 μL total reaction volume. The primer pair used for amplification was 5′-AAAGGGCCCTGACAACTCTTT-3′, GAPDH sense and 5′-TCAGTCTGAGGAGAACATACCA-3′ antisense with 400 bp expected product size (Eurofins Scientific). The concentration of each primer in the total volume of sample was as recommended by the enzyme manufacture. The reaction also contained metal ions at the concentrations shown in the experimental results.

All sample analysis was performed on a StepOnePlus™ Real-Time PCR System (Applied Biosystems) under the following cycling mode: one initial cycle at 50 °C for 2 min, one cycle at 95 °C for 2 min, followed by 40 cycles of denaturation at 95 °C for 15 s, annealing at 60 °C for 15 s and extension at 72 °C for 1 min.

### Effect of metal ions on various DNA polymerases

Tested polymerases included KOD polymerase from KOD Hot Start DNA Polymerase kit (Merck KGaA), *Taq* polymerase from MyTaq™ Red Mix (Bioline) and Q5 DNA polymerase from Q5® High-Fidelity DNA Polymerase (New England Biolabs). All the reactions were performed following the manufacturer’s recommended protocols. The primers used for amplification were the GAPDH primers as described above. The final concentration of primers for each polymerase was adjusted as per the manufacturer’s recommendations as follows: 0.3 μM for KOD polymerase reactions, 0.5 μM for Q5 polymerase, 0.3 μM for Taq polymerase. Each sample contained 1 ng of template DNA (TaqMan™ Control Genomic Human DNA, Thermo Fisher Scientific). PCR amplification was performed in a Veriti™ 96-Well Thermal Cycler (Applied Biosystems®) under the following cycling conditions: (a) for Q5 polymerase: one initial cycle at 98 °C for 30 s, 30 cycles at 98 °C for 10 s, at 63 °C for 30 s and at 72 °C for 30 s, followed by final cycle at 72 °C for 30 s; (b) for KOD polymerase: one initial cycle at 95 °C for 2 min, 30 cycles at 95 °C for 20 s, at 63 °C for 30 s and at 72 °C for 30 s; (c) for Taq polymerase: initial cycle at 95 °C for 1 min, 30 cycles at 95 °C for 15 s, at 63 °C for 15 s and at 72 °C for 10 s. The results of amplification were visualised on 1% agarose gel.

### Removing calcium inhibition

Samples containing five different concentrations of calcium chloride (1 to 5 mM) were treated with EGTA (ethylene glycol-bis(2-aminoethylether)-N,N,N′,N′-tetraacetic acid, Sigma-Aldrich) at a final concentration equal to that of the added calcium chloride. Samples were amplified using KOD Hot Start DNA Polymerase or amplified in qPCR reaction mix using Applied Biosystems™ PowerUp™ SYBR™ Green Master Mix. The primers and cycling conditions were as described above for KOD polymerase samples. TaqMan™ Control Genomic Human DNA was used as template at either 1 ng for standard PCR or 5 ng DNA for qPCR. The amplification products were visualised by electrophoresis on a 1% agarose gel or analysed by the StepOne software respectively.

## Results and discussion

### Effect of metal ions on real-time PCR

**Fig. 1:**
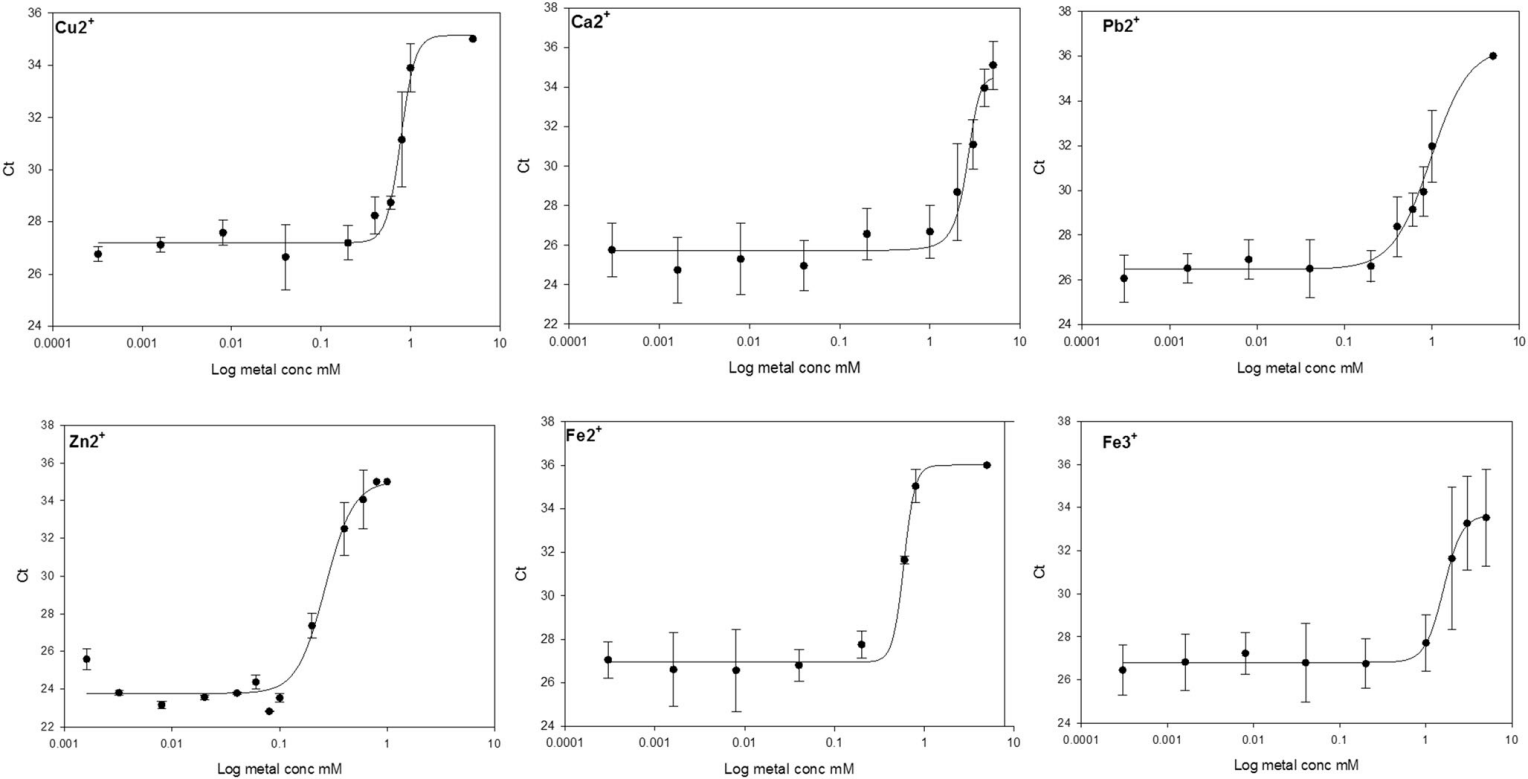
Representative data for the inhibition of qPCR by the metal ions shown in the figure over a 4 log concentration range. PCR conditions are as described in “Materials and methods” with 5 ng input DNA per reaction. The data is plotted as mean and standard deviation Ct value from triplicate qPCR reactions and a four-parameter logistic curve fitted by Sigma Plot non-linear regression to calculate IC50 values

The degree of PCR inhibition by metal ions was determined by assessing the qPCR cycle threshold (Ct) values in the presence of added metal ions over a 4-log concentration range (0.002 to 5 mM) as detailed in “Materials and methods”. Amplification of the expected product was checked by agarose gel electrophoresis (Supplementary data). No DNA was detectable by gel electrophoresis (Supplementary data) in any samples with a Ct value greater than 35. Samples reported by the one plus software as undetected were assigned a Ct value of 36. The results presented in Fig. 1 show representative inhibition curves for copper, calcium, iron(II), iron(III), zinc and lead. The plots were obtained by non-linear regression of a four-parameter logistic curve to the data using Sigma Plot 14 software and an IC50 (concentration of metal giving 50% inhibition of PCR) value obtained for each metal. The IC50 values obtained for all the tested metals are shown in Table 1. These results suggest that of the tested metals, zinc, tin, copper and iron(II) have the greatest potential to cause PCR inhibition and as a result lead to unsuccessful DNA analysis. It is interesting to note that copper, zinc and occasionally tin are the main components of brass used in cartridge cases which may go some way in explaining the poor recovery of DNA from ammunition [11, 12].

**Table 1:** IC50 values for the inhibition of qPCR as determined by Sigma Plot software from the non-linear regression analysis of inhibition curves. Representative data is shown in Fig. 1

Metal	IC50 mM	sd
Al	2.79	0.49
Ca	2.56	0.21
Fe III	1.60	0.08
Ni	2.27	0.09
Cu	0.77	0.04
Fe II	0.59	0.02
Pb	0.96	0.09
Sn	0.31	0.17
Zn	0.26	0.02

In a study testing the effect of metals on Quantifiler® Human DNA Quantification Kit conducted by Combs et al. [1], it indicated that aluminium was the most potent inhibitor, with 50% inhibition obtained around 100 μM final concentration in the PCR reaction as estimated from their published figures. In our study, the IC50 value for aluminium is 2.79 mM. Conversely in the case of calcium, we observed an IC50 of 2.56 mM whereas Combs et al. [1] observed no inhibition up to 1.5 mM which is the maximum final concentration of any metal they used. It should be noted that the data presented by the authors in Combs et al. [1] (Fig. 1) plot the concentration of metal in the stock solution of DNA and not the concentration of metal they added to the PCR reactions. The discrepancies between these results and ours are most likely due to the differences in the PCR methodology used. In Combs et al. [1], the Quantifiler kit uses a Taqman probe displacement assay which contains a reporter probe attached to a DNA minor groove binder protein. This gives another point of potential inhibition by metal interaction with the minor groove binder protein which could explain the general observation that the inhibitory effects observed occurred at lower concentrations than in our study, reflecting an inhibition of the reporter mechanism rather than actual inhibition of the PCR reaction. Given that the amplification of forensic samples does not involve the presence of reporter Taqman probes, we would argue that our data reflects more closely the reactions occurring in the normal profiler reactions. Additionally, as demonstrated in the second part of our study, different master mixes and polymerases can have different susceptibility to potential inhibitors.

### Effect of metal ions on various DNA polymerases

**Fig. 2:**
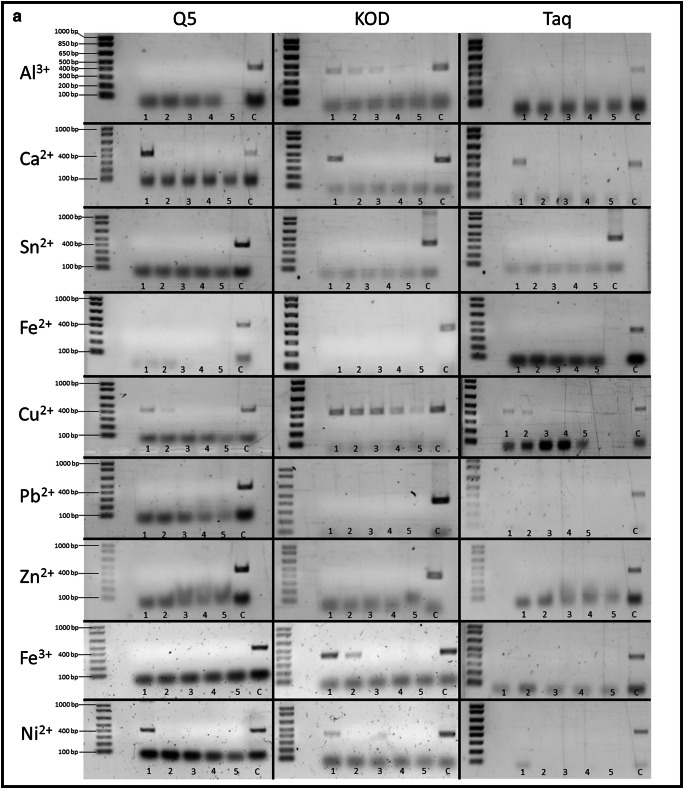

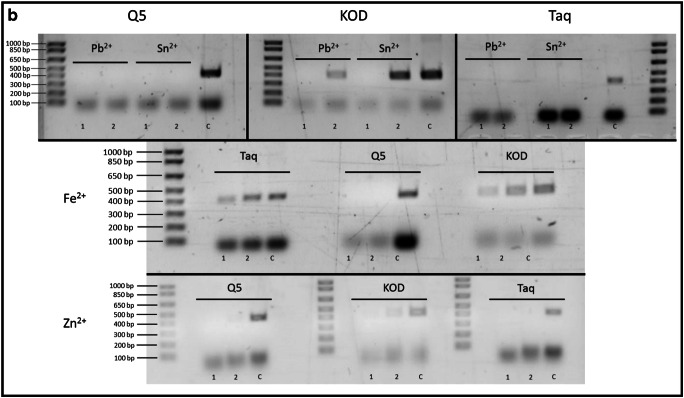
**a** Differential effect of metal ions on the activity of PCR polymerases Q5, KOD and Taq. PCR conditions are as described in “Materials and methods”. Lanes 1–5 show PCR products from reactions containing metal ions at concentrations of 1–5 mM respectively. C represents control reactions with no metal additions. **b** Metal ions which showed complete inhibition of PCR at the concentrations shown in **a** were further tested at lower metal concentrations of 0.5 mM (1) and 0.25 mM (2) and no metal (C). All other parameters were as for **a**

Three commonly used DNA polymerases Taq, Q5 and KOD were tested for their susceptibility to inhibition by metal ions. These enzymes were chosen as they are divergent in origin and not just variant of standard Taq polymerase. Taq and KOD polymerases are from different bacterial phyla and therefore diverse in sequence and structure and Q5 which again is completely different in origin in that it is a synthetic genetic construct containing thermostable DNA polymerase with 3′ → 5′ exonuclease activity, fused to a processivity-enhancing Sso7d domain. Initially, each set of reactions was treated with the same range of metal ion concentrations of 1 to 5 mM. Those samples that failed to amplify were tested again at lower concentrations of 0.5 and 0.25 mM. Performance of each polymerase was assessed by the presence, absence and intensity of the expected DNA product band on an agarose gel of the PCR reaction. The data presented in Fig. 2 shows the effect of metal ions on the tested polymerases Taq, Q5 and KOD. From the data presented, we can see that KOD polymerase is the most resistant to inhibition by aluminium ions with activity being detected at up to 4 mM aluminium. In the case of calcium, Q5 appears to be the most resistant to inhibition but none of the enzymes is active at calcium concentrations greater than 1 mM. KOD polymerase is also most resistant to copper inhibition with faint activity being detected at 5 mM copper whereas Q5 and Taq show no activity at concentrations greater than 1 mM. For iron(III), again KOD is the most resistant to inhibition still showing activity at 2 mM metal. In the case of nickel, Q5 appears to be the most resistant but no enzyme was active in greater than 1 mM metal. None of the enzymes was active in the presence of iron(II), lead, zinc or tin at 1 mM concentrations. They were subsequently tested at the lower concentrations 0.5 and 0.25 mM as shown in Fig. 2b. The results show that, unlike Taq and Q5, KOD can tolerate both tin and lead at 0.25 mM and that both Taq and KOD can tolerate iron(II) at 0.5 mM. None of the tested enzymes tolerates zinc very well with only KOD showing a trace of activity at 0.25 mM. These results demonstrate that contamination by metals may be, at least partially, overcome by the choice of enzyme used in the amplification reaction. The results also show that the KOD polymerase is remarkably tolerant of contaminating metal ions compared with Q5 and standard Taq polymerases.

When extracting DNA from a biological sample, purification is always part of the standard procedure [34] and since purification itself can remove many inhibitors and impurities from a DNA sample, in some cases inhibition may not even be an issue when dealing with a crime scene sample. Additionally, metals may not affect encapsulated DNA as they do naked DNA. In its encapsulated form, DNA benefits from a protective barrier provided by enclosing it in the cell and nuclear structure [35] and additional protection comes from DNA being tightly bound to histones [36]. However as suggested by Bille et al., poor DNA recovery from metal objects may be caused by more than just inhibition of PCR during amplification [29]. In their study, the authors indicate that with some metals, DNA loss may occur at much earlier stages, before the recovery process even begins. Their study used the antibacterial properties of copper to investigate any potential cell damage when in contact with the copper surface, speculating that one of the reasons behind poor DNA recovery from certain types of metal could be the fact that there may not be much DNA left on the surface due to the damaging effect of copper. In our study, the metals were added in solution just prior to amplification to minimise any potential reaction with the DNA template. We cannot, however, be certain that no such interaction took place but note that in our protocol the metals are present as ions in solution rather than as native metal surfaces. DNA degradation due to prolonged exposure to copper combined with inhibitory properties of Cu metal ions clearly has the potential for a negative impact on DNA analysis, as poor results of DNA recovery from copper surfaces have also been demonstrated in other studies [20, 33]. Excessive amounts of copper have been shown to cause damage to human DNA in vitro [37–39] while in vivo, the body possesses a number of efficient mechanisms for copper homeostasis [37].

Our study demonstrates (Fig. 2) that PCR reactions are not equally affected by the same inhibitors, a situation that has been observed before [40]. Of the three tested polymerases, KOD turned out to be the most resistant to inhibitory properties metal ions. KOD polymerase is known for its high-fidelity, thermostable proofreading capacity and high processivity [41]. This is not the first time KOD has proven to be the best polymerase against challenging specimens. In previous studies, KOD has been shown to be the most suitable polymerase when dealing with difficult samples such as specimens from heavy metal polluted soil, also showing great resistance to humic acid [42]. Moreover, in comparison with Taq polymerase in PCR-mass spectrometry–based analyses, amplification with KOD polymerase resulted in a 2 to 3 fold increase of PCR product [43].

### Removing calcium inhibition

Despite decalcification being part of the process of DNA extraction from bones, contamination of DNA samples with calcium ions is still a possibility and can lead to a challenging DNA analysis [44]. The current methods of removing calcium can result in DNA losses during purification due to the extra steps involved. When dealing with non-forensic specimens, one of the most common techniques used to overcome the impact of an unknown inhibitor is diluting the sample [6], by so doing the concentration of inhibitors is reduced relative to the DNA target. This method is known to produce satisfactory results when applied to food and environmental samples [6]. Nonetheless, this method would not be appropriate for the majority of forensic samples as, in many cases, the volume of sample, and therefore available DNA is already very limited. Diluting an already low concentration of a DNA sample may result in DNA below the limit of detection.

**Fig. 3:**
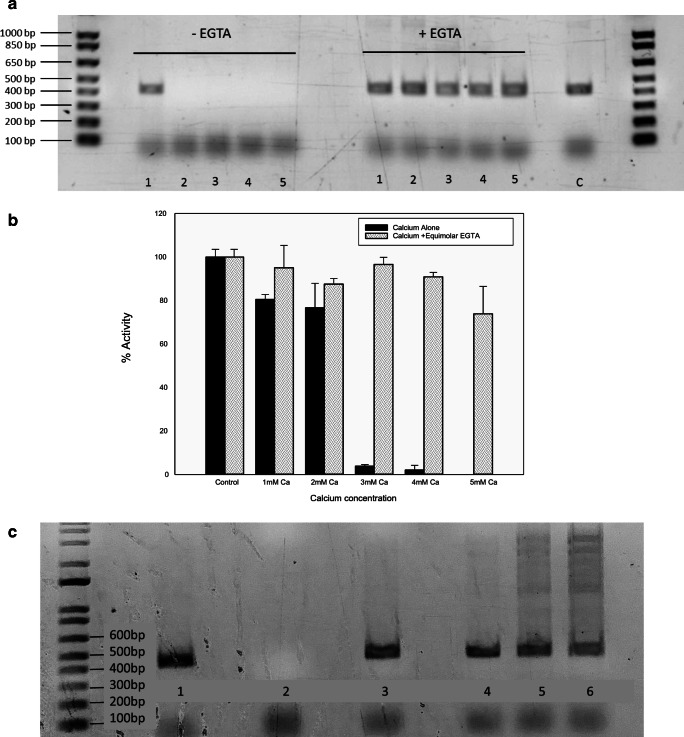
**a** Agarose gel electrophoresis of standard PCR reactions with increasing concentrations of calcium, 1–5 mM as numbered, showing inhibition of PCR at 2 mM calcium and reversal of inhibition in at all calcium concentrations in the presence of an equimolar amount of EGTA. Lane C is a control reaction containing no added calcium. All other parameters were as described in “Materials and methods”. **b** SYBR Green qPCR inhibition by calcium, 1–5 mM, and reversal by the addition of an equimolar amount of EGTA. Data is shown as mean and standard deviation of triplicate reactions normalised to 100% activity of the control reaction containing no calcium. Cleary demonstrating the reversal of inhibition by the addition of EGTA to the reactions. All other parameters are detailed in “Materials and methods”. **c** Effect of increasing Mg^2+^ in addition to reversal of calcium inhibition of standard PCR inhibition by EGTA. (1) Control with no calcium or extra Mg^2+^ addition, (2) addition of 2 mM calcium giving complete inhibition, (3) addition of 2 mM calcium with 2 mM EGTA reversing inhibition, (4–6) as for (3) with Mg^2+^ added at 0.5, 1.0 and 1.5 mM respectively showing increasing non-specific amplification with the increasing Mg^2+^ concentration

The data presented in Fig. 3a shows that the inhibition of a standard PCR reaction due to calcium can be reversed by the addition of the calcium chelator, EGTA, as assessed by agarose gel electrophoresis. The data shows that in the presence of 1 to 5 mM calcium, no DNA amplification can be detected; however, when an equimolar amount of EGTA is added to the reaction, amplification can be detected at all calcium concentrations. Quantitative data from qPCR reactions containing calcium or calcium plus EGTA is shown in Fig. 3b again demonstrating that the calcium inhibition can be overcome by the addition of EGTA to the reaction although the qPCR reaction seems to be inherently more tolerant of calcium in that product is produced in the presence of 2 mM calcium whereas in the standard PCR reaction, no product was visible on the gel at 2 mM calcium. The reversal of calcium inhibition is due to the differential affinity of EGTA for calcium over magnesium [45, 46] effectively removing the calcium while leaving the magnesium relatively unaffected which is essential for the PCR reaction. Increasing the concentration of magnesium (Mg^2+^) to levels well above those required for a standard PCR run has been proven to reverse the inhibitory effects of calcium ions [3]. Unfortunately, there are many drawbacks involved in increasing concentrations of Mg^2+^ in a PCR reaction. Too much magnesium can decrease fidelity and specificity of DNA polymerase as well as interfere with complete denaturation of DNA strands during amplification [47]. In addition, increased levels of Mg^2+^ can lead to primers annealing to incorrect sites of DNA template and consequently result in appearance of unwanted PCR products [47]. Additional experiments were carried out where the EGTA addition was supplemented with extra magnesium but this was shown to have no significant effect on the amount of the desired product produced but as noted above there was an increase in the amount of non-specific amplification as shown in Fig. 3c.

## Conclusions

The complex interactions between metals and DNA remain a source of many issues involved in DNA analysis. Despite many studies describing undesirable effects of metal ions on DNA and amplification process, there has been little research into ways of enhancing recovery or processing of DNA samples that have come into contact with a metal source. In this study, we demonstrate the effect of metal ions on PCR efficiency and how the choice of DNA polymerase may lead to a better outcome of PCR amplification in the presence of inhibitors.

We have found that zinc, tin, iron(II) and copper ions have the greatest potential to interfere with amplification, with IC50 values of 0.26, 0.31, 0.59 and 0.77 mM respectively. Aluminium and calcium turned out to have the least inhibitory effect with IC50 values of over 2 mM. Out of three commonly used DNA polymerases, KOD turned out to be the most resistant to metal inhibition. This short study suggests that relying on one type of polymerase in commercial forensic kits may not always guarantee the best results. We also demonstrated a simple and non-destructive method of preventing inhibitory effects of calcium ions in a PCR reaction by addition of the calcium chelator EDTA.

There is clearly a need for deeper understanding of mechanisms leading to metal interactions with DNA and polymerases in the context of forensic analysis. It is important to determine whether the problems occur due to failure to extract DNA from samples recovered from metal surface or whether the inhibitory substances are being extracted alongside the DNA sample. Here we focused exclusively on the amplification aspect, but further investigation is recommended to examine other variables. Identifying the source of the problem will certainly help with minimizing the effects of metals on DNA samples.

## Electronic supplementary material

ESM 1 (DOCX 2363 kb)
