# Supplementary material for: Impact of metal ions on PCR inhibition and RT-PCR efficiency
Source: Int J Legal Med. 2020 Jul 3;135(1):63–72. doi: 10.1007/s00414-020-02363-4 (PMC7782418; doi:10.1007/s00414-020-02363-4)

**Supplementary Data**

Gel electrophoresis analysis of qPCR samples from metal inhibition studies confirming product identity.


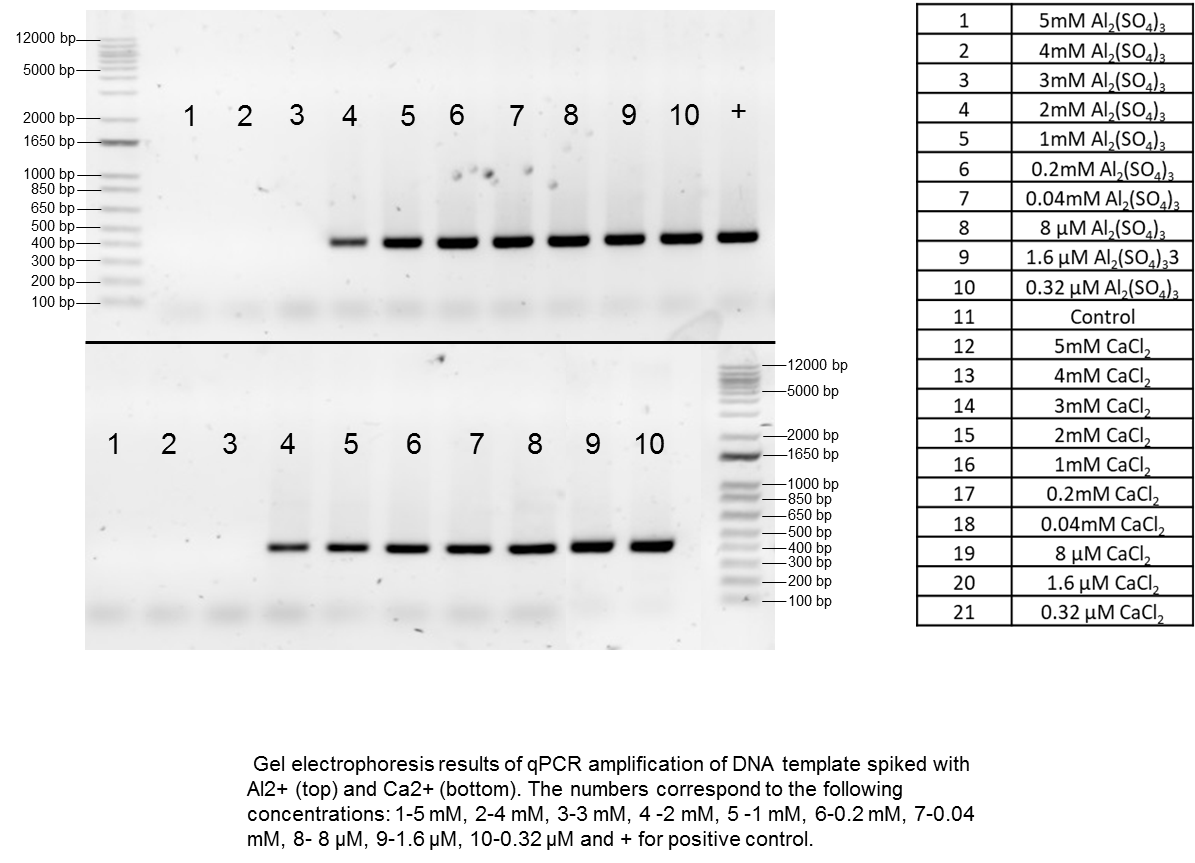


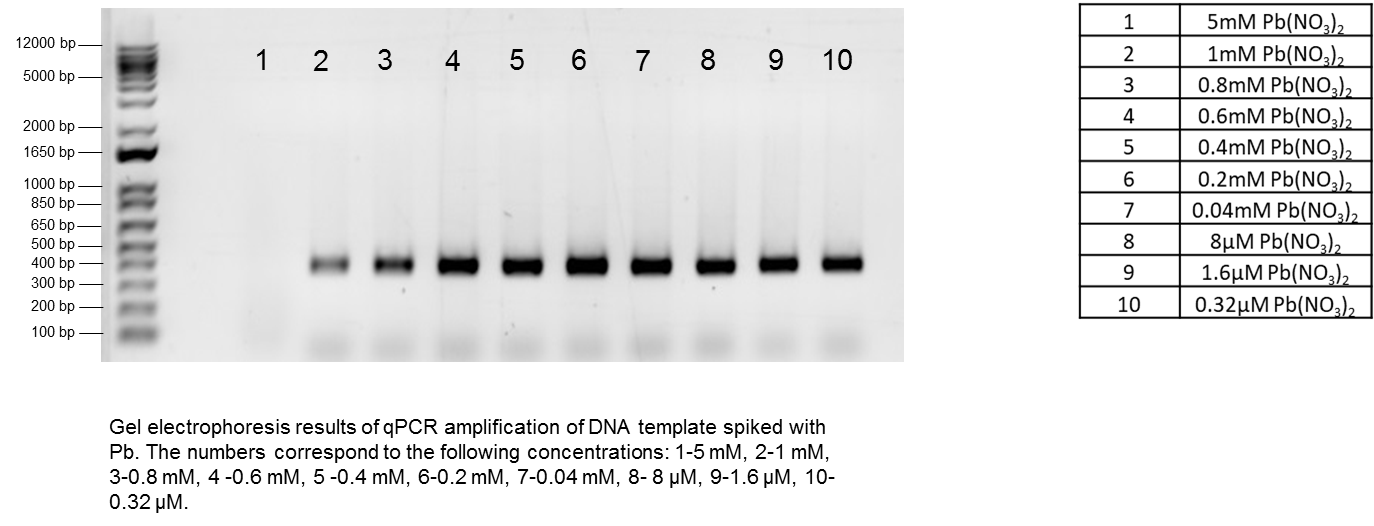


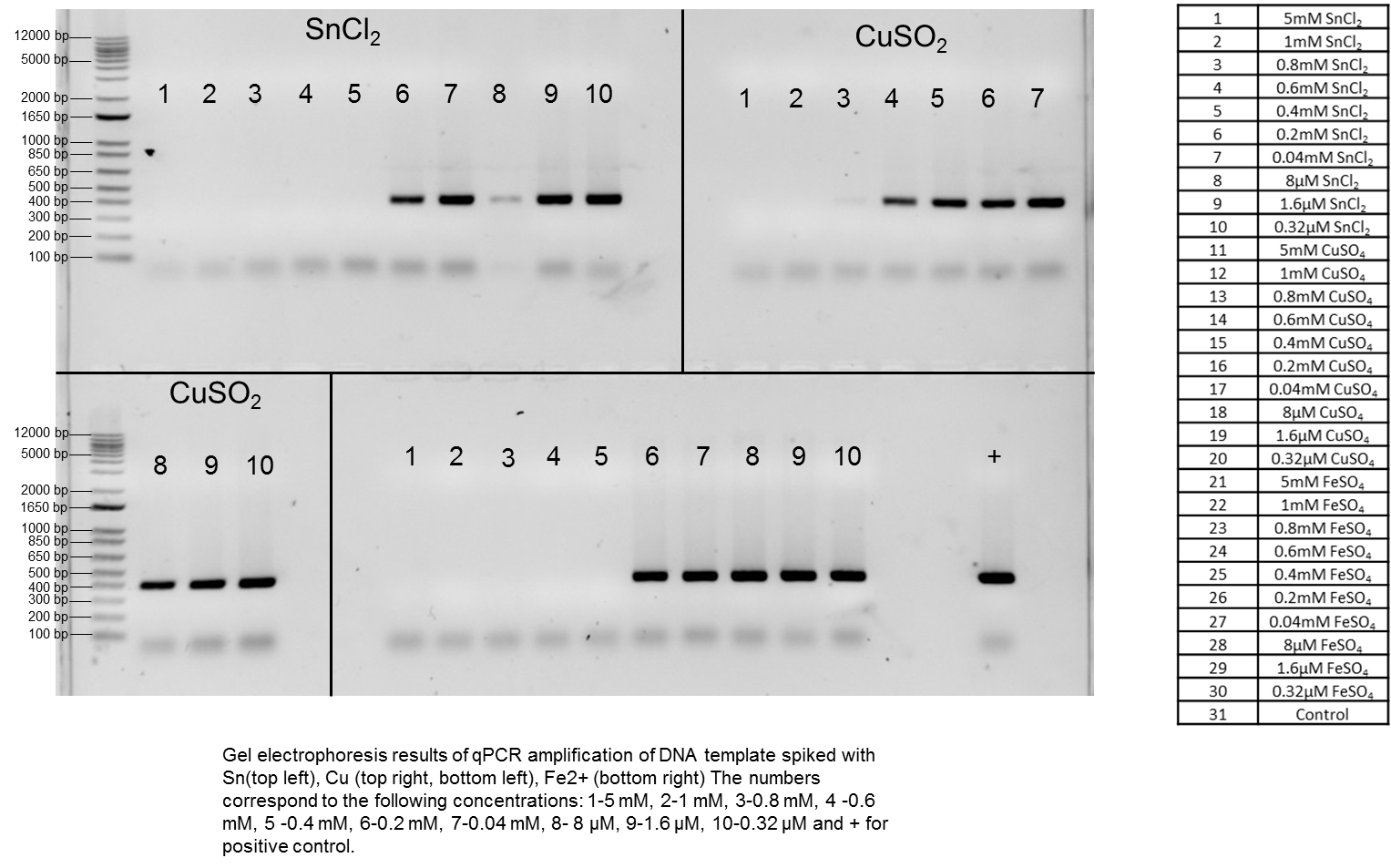


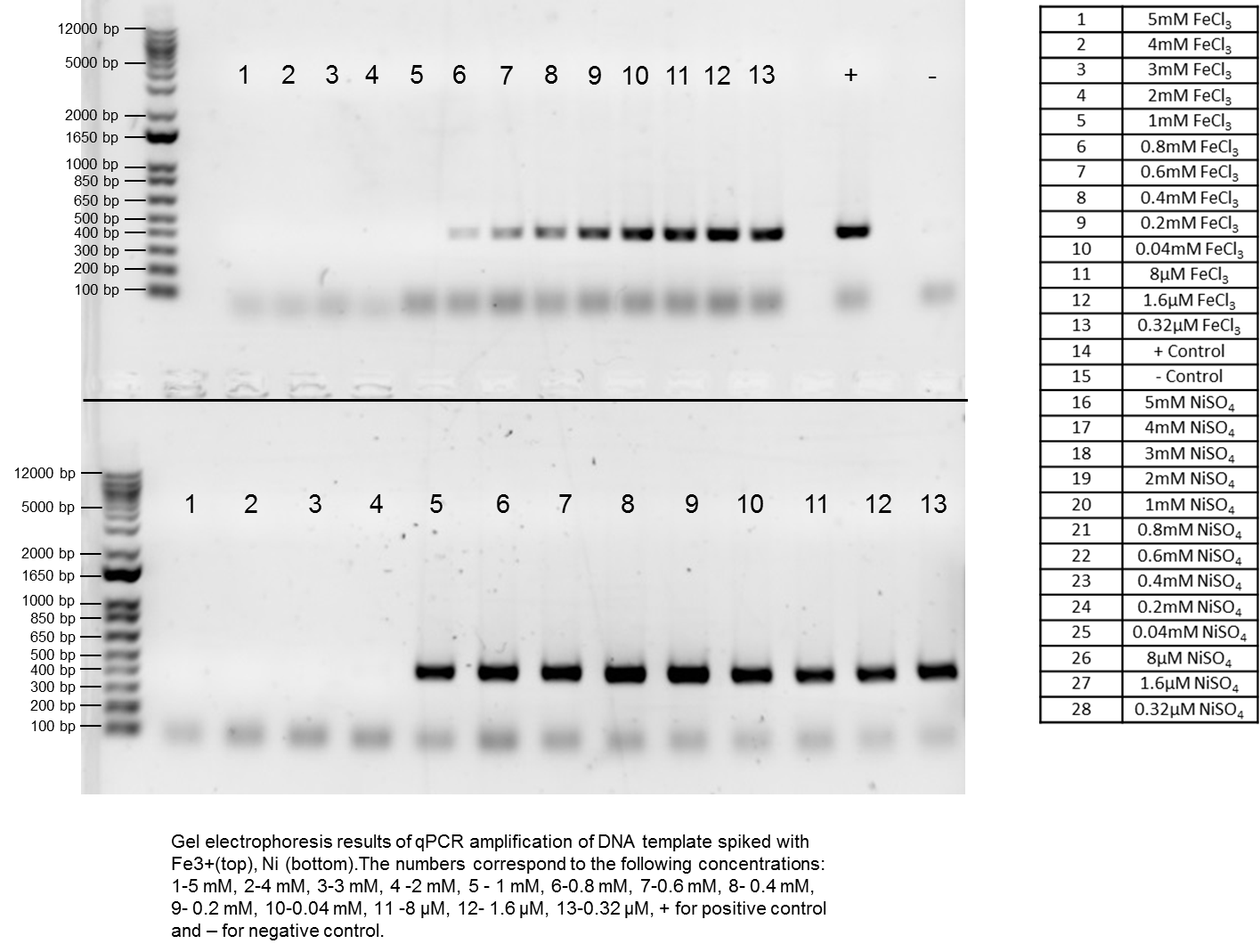


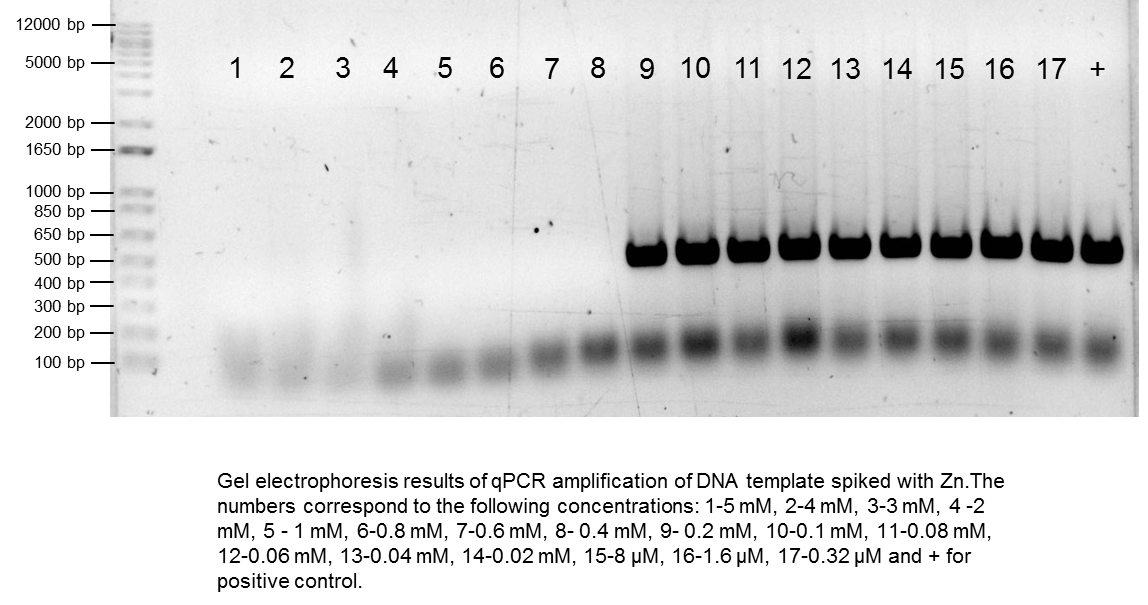

Supplement: Supplementary file 1 — (DOCX 2363 kb) [file 414_2020_2363_MOESM1_ESM.docx]
